# Bronchoscopic Cryobiopsy for the Diagnosis of Diffuse Parenchymal Lung Disease

**DOI:** 10.1371/journal.pone.0078674

**Published:** 2013-11-12

**Authors:** Jonathan A. Kropski, Jason M. Pritchett, Wendi R. Mason, Lakshmi Sivarajan, Linda A. Gleaves, Joyce E. Johnson, Lisa H. Lancaster, William E. Lawson, Timothy S. Blackwell, Mark P. Steele, James E. Loyd, Otis B. Rickman

**Affiliations:** 1 Division of Allergy, Pulmonary and Critical Care Medicine, Department of Medicine, Vanderbilt University School of Medicine, Nashville, Tennessee, United States of America; 2 Department of Pathology, Microbiology and Immunology, Vanderbilt University School of Medicine, Nashville, Tennessee, United States of America; 3 Departments of Cell and Development Biology and Cancer Biology, Vanderbilt University School of Medicine, Nashville, Tennessee, United States of America; 4 Department of Thoracic Surgery, Vanderbilt University School of Medicine, Nashville, Tennessee, Unites States of America; 5 Department of Veterans Affairs Medical Center, Nashville, Tennessee, United States of America; University of Washington, United States of America

## Abstract

**Background:**

Although in some cases clinical and radiographic features may be sufficient to establish a diagnosis of diffuse parenchymal lung disease (DPLD), surgical lung biopsy is frequently required. Recently a new technique for bronchoscopic lung biopsy has been developed using flexible cryo-probes. In this study we describe our clinical experience using bronchoscopic cryobiopsy for diagnosis of diffuse lung disease.

**Methods:**

A retrospective study of subjects who had undergone bronchoscopic cryobiopsy for evaluation of DPLD at an academic tertiary care center from January 1, 2012 through January 15, 2013 was performed. The procedure was performed using a flexible bronchoscope to acquire biopsies of lung parenchyma. H&E stained biopsies were reviewed by an expert lung pathologist.

**Results:**

Twenty-five eligible subjects were identified. With a mean area of 64.2 mm^2^, cryobiopsies were larger than that typically encountered with traditional transbronchial forceps biopsy. In 19 of the 25 subjects, a specific diagnosis was obtained. In one additional subject, biopsies demonstrating normal parenchyma were felt sufficient to exclude diffuse lung disease as a cause of dyspnea. The overall diagnostic yield of bronchoscopic cryobiopsy was 80% (20/25). The most frequent diagnosis was usual interstitial pneumonia (UIP) (n = 7). Three of the 25 subjects ultimately required surgical lung biopsy. There were no significant complications.

**Conclusion:**

In patients with suspected diffuse parenchymal lung disease, bronchoscopic cryobiopsy is a promising and minimally invasive approach to obtain lung tissue with high diagnostic yield.

## Introduction

There are over 150 recognized causes of diffuse parenchymal lung disease (DPLD, also known as “interstitial lung disease”, ILD). While in certain circumstances, classic clinical features and radiography are sufficient to establish a diagnosis [1], frequently additional testing including bronchoscopy and/or surgical lung biopsy are required. The ATS/ERS guidelines for diagnosis and treatment of idiopathic pulmonary fibrosis (IPF), the most common and most severe form of idiopathic ILD, recommend surgical lung biopsy if classic radiographic criteria are not found [1]. In two recent clinical trials, 22–54% of IPF patients had undergone surgical lung biopsy to establish the diagnosis [2], [3], suggesting that in spite of advances in imaging of DPLDs, a substantial number of patients require biopsy.

Surgical lung biopsy requires endotracheal intubation, general anesthesia, chest tube placement and typically hospitalization for several days. There is also risk of prolonged air leak and/or bronchopleural fistula requiring prolonged hospitalization and/or reoperation, and low but significant risk of mortality [4], [5]. Given these potential risks and complications, there is need for a less invasive technique for lung biopsy.

Bronchoscopy is commonly employed in the diagnostic evaluation of DPLD, however for most DPLD’s, transbronchial biopsy (TBBx) specimens have low diagnostic yield. In a large series reviewing 801 DPLD patients who underwent transbronchial biopsies, less than 1/3 established a specific diagnosis, and of those cases nearly all were malignant or infectious in origin [6]. Specifically among patients with idiopathic ILD, the yield appears to be low. In one series of patients ultimately found to have IPF, only 3/32 specimens met usual interstitial pneumonia (UIP) criteria [7]. In several other series, 30–34% of transbronchial biopsies had at least 1 feature consistent with UIP/IPF but were overall inconclusive [8], [9].

Flexible cryo-probes have been used for bronchoscopic procedures including endobronchial biopsy and tumor ablation with success. Recently, these probes have been employed for peripheral lung biopsy in several small series and have been shown to be safe [10], [11], [12]. In this study, we sought to examine the diagnostic yield of bronchoscopic cryobiopsy among patients with DPLDs and characterize cryobiopsy specimens in these patients. Some of these results have been presented previously in abstract form at the 2013 American Thoracic Society International Conference, May 19, 2013 [13].

## Materials and Methods

### Subjects

This study was approved by the Vanderbilt University Institutional Review Board (IRB # 121556) with a waiver of informed consent as for this study existing data were analyzed in a deidentified manner. Subjects were identified by a retrospective chart review of all patients who had undergone bronchoscopic cryobiopsy at Vanderbilt University Medical Center for the diagnosis of DPLD from January 1, 2012 through October 29, 2012. All subjects had suspected interstitial lung disease based on clinical information, serology testing, and high-resolution computed tomography (HRCT) scans with atypical features requiring lung biopsy. The decision to perform bronchoscopic cryobiopsy was made by the patient’s attending physician as a component of the clinical evaluation of the patient.

Safety outcomes are also reported for a cohort of research subjects that were identified from an ongoing study of asymptomatic family members at-risk for familial interstitial pneumonia (FIP) [14] (Vanderbilt University IRB #87780). Written informed consent was obtained from subjects prior to enrollment. Eligible subjects were >40 years of age and had a first degree relative with FIP. After enrollment in the study, eligible subjects were administered a modified American Thoracic Society Diffuse Lung Disease 78 (ATS-DLD78) questionnaire. Subjects with a Dyspnea Score <3 were considered asymptomatic as this had previously been shown to be predictive of pulmonary function test abnormalities [15]. All subjects then underwent HRCT with prone positioning as per the study protocol.

### Bronchoscopy and Biopsy

**Figure 1:**
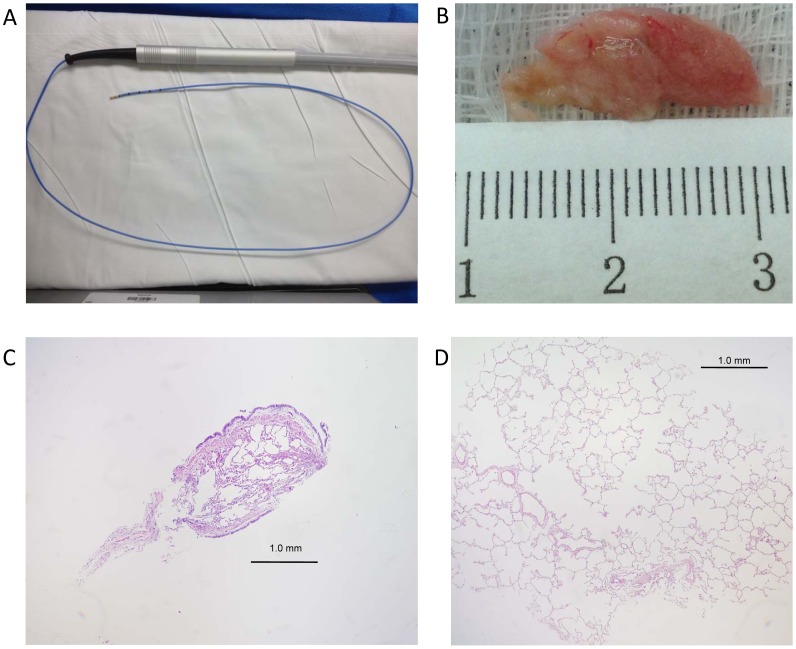
Bronchoscopic cryoprobe and low power images of biopsies demonstrate cryobiopsies are larger than typical forceps biopsies. (A) Bronchoscopic cryoprobe. (B) Gross pathology of a representative cryobiopsy specimen measuring 19 mm in length. (C) Typical transbronchial forceps biopsy. (D)) Normal bronchoscopic cryobiopsy sample. All images were taken at low power (40×).

Bronchoscopies were performed by an attending interventional pulmonologist with standard monitoring and sedation procedures in the bronchoscopy suite. Seven days prior to the procedure, patients were required to stop all anti-platelet agents: aspirin, clopidogrel and herbal supplements. Patients were administered intravenous conscious sedation by certified registered nurse anesthetist under the direction of an anesthesiologist. A Portex cuffed 8.5 wire-spiral endotracheal tube was positioned in the airway with the bronchoscope (Olympus XT180, Olympus America Inc) with the cuff deflated to permit spontaneous breathing. This allowed rapid re-entry into the airway after the biopsy was done without having to re-navigate through the nose or mouth. Bronchoscopic cryobiopsy was targeted to the areas of abnormality seen on HRCT. Among research subjects with no HRCT abnormalities, biopsies were performed in the posterior and lateral segments of the right lower lobe. The Erbe 1.9 mm cryoprobe (Erbe Elektromedizin GmBH) (Figure 1A) was advanced through the working channel and into the distal parenchyma under fluoroscopic guidance. The probe was withdrawn ∼2 cm from the point resistance was met. Then on exhalation a 4-second freeze time was applied and then the probe and bronchoscope were removed en-bloc from the airway, the probe with the frozen biopsy specimen was not pulled into the bronchoscope. The extended probe and frozen specimen was submerged into room temperature saline to speed thawing and removal of specimen. The probe was then withdrawn from working channel and the bronchoscope was then reintroduced into the airway to clear bleeding and then wedged into the biopsied subsegment for three minutes. Two biopsies total, each from a different segment were taken for each procedure. The specimens were removed immediately from the saline and placed in formalin.

### Clinical Parameters

Clinical data were extracted by retrospective chart review independently by two of the authors; any disagreement was resolved by consensus discussion.

### Biopsy Specimens

Biopsy specimens were fixed in 10% formalin and embedded in paraffin. Hematoxylin and eosin (H&E) stained slides were reviewed by the standard protocol of the Vanderbilt clinical pathology laboratory; research samples were processed in an identical manner. Clinical specimens were reviewed under standard clinical care and research specimens were reviewed by an expert lung pathologist who was blinded to subject information.

### Diagnostic Yield

Clinical parameters, radiography and biopsy results were reviewed independently by two of the authors who separately recorded a diagnosis for each clinical subject. Biopsies were considered “diagnostic” if after reviewing the clinical information, radiology and pathology both reviewers and the treating clinician reached a uniform diagnosis and no further evaluation (i.e. surgical lung biopsy) was pursued. Disagreement was resolved by consensus discussion between the reviewers and an expert lung pathologist; there was disagreement regarding 2/25 clinical biopsies, both of which were ultimately deemed “non-diagnostic.”

## Results

During the study period, 25 subjects underwent cryobiopsy for evaluation of DPLD. An additional 12 asymptomatic subjects at-risk for FIP underwent cryobiopsy as per the study protocol. Subject characteristics are summarized in Table 1. In total, 73 cryobiopsies were performed during the study period. Biopsy characteristics of clinical cryobiopsies are summarized in Table 2. In 7 patients, both upper and lower lobes were sampled; in the remaining 18 subjects, biopsies were taken from multiple segments of the lower lobes. Alveolated lung was present in 24/25 clinical biopsy samples. Typical examples of a forceps biopsy and cryobiopsy are shown in Figure 1. Mean cryobiopsy length was 8.7 mm (range 6–31 mm), and mean biopsy area was 64.2 mm^2^ (range 1.5–136.7 mm^2^), which is substantially larger than 5–15 mm^2^ reported in several recent studies of transbronchial forceps biopsy [10], [12].

**Table 1:** Subject characteristics.

	Clinical Subjects	Research Subjects
	(n = 25)	(n = 12)
Age	57.1 (27–75)	53.8 (42–61)
Sex (male)	13 (52%)	2 (17%)
Outpatient	21 (84%)	12 (100%)
Clinical/Radiographic Diagnosis		
ILD	19 (76%)	
Bronchiolitis	6 (24%)	
Family History of ILD	3 (12%)	12 (100%)
Pulmonary Function Tests		
FEV1 (L)	2.44 (0.76)	
FEV1% predicted	76.4 (16.4)	
FVC (L)	3.09 (1.02)	
FVC % predicted	75.3 (17.0)	
TLC (L)	4.80 (1.68)	
TLC % predicted	75.5 (19.1)	
DLCO (ml/min/mmHg)	15.3 (5.5)	
DLCO % predicted	66.2 (25.3)	

Mean (range) or number (percentage) are presented as appropriate for demographic information. Pulmonary function tests are reported as mean (standard deviation). DLCO is reported corrected for hemoglobin when available.

**Table 2:** Biopsy characteristics.

Number	2 (1–2)
Biopsy location	
RUL and RLL	6
RUL and LLL	1
RLL	8
LLL	10
Length (mm)	8.7 (5.1)
Area (mm^2^)	64.2 (41.4)
Alveoli present	24 (96%)
Diagnostic	20 (80%)
Specific DPLD	19 (76%)
Normal	1 (4%)
Nonspecific findings	5 (20%)
Surgical Biopsy	3 (12%)

Data are presented as mean and range or mean and standard deviation where appropriate. Biopsy location is reported per subject. Percentages are calculated relative to total number of clinical biopsy subjects (n = 25).

**Figure 2:**
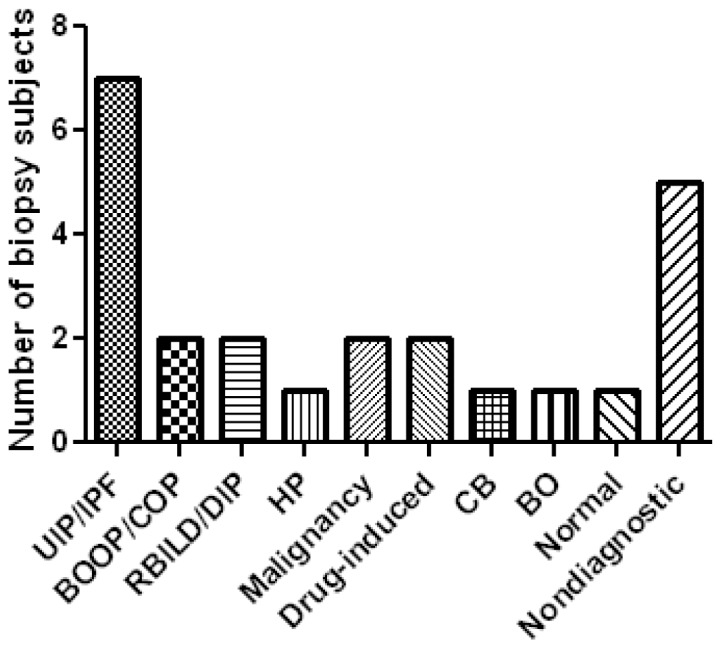
Distribution of diagnoses made by bronchoscopic cryobiopsy in 25 clinical subjects. Abbreviations: UIP/IPF, usual interstitial pneumonia/idiopathic pulmonary fibrosis; OP, organizing pneumonia; RBILD/DIP, respiratory bronchiolitis-interstitial lung disease/desquamative interstitial pneumonia; HP, hypersensitivity pneumonitis; CB, constrictive bronchiolitis.

**Figure 3:**
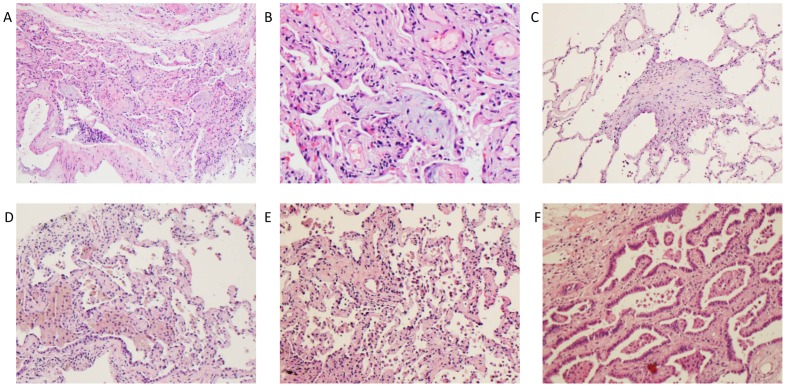
Bronchoscopic cryobiopsy identifies characteristic histopathology from multiple DPLDs. Representative hematoxylin and eosin stained cryobiopsies from subjects with cryptogenic organizing pneumonia (A) 100x and (B) 400×, (C) bronchiolitis obliterans (200×), (D–E) respiratory bronchiolitis-interstial lung disease/desquamative interstitial pneumonia(100×) and (F) adenocarcinoma with lepidic spread, previously bronchoalveolar cell carcinoma (100×). Power reported as original magnification.

**Figure 4:**
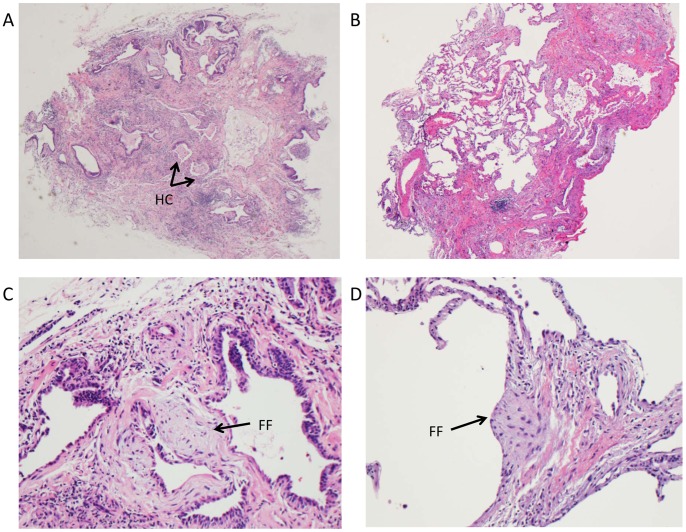
Cryobiopsy demonstrates typical features of UIP/IPF. Representative cryobiopsy samples from subjects with UIP demonstrating (A) dense scar with microscopic honeycomb cysts (HC), (B) Temporal heterogeneity, and (C–D) Fibroblastic foci (FF). A and B original magnification 40×, C-D 400×.

Among clinical biopsies a specific clinical-pathologic diagnosis was achieved in 19/25 cases. In one case, normal lung tissue obtained by cryobiopsy was felt sufficient to exclude DPLD as a cause of dyspnea. Three subjects with nondiagnostic cryobiopsies were subsequently referred for surgical lung biopsy, two of which were diagnostic, one demonstrating UIP and another cryptogenic organizing pneumonia (COP). Among the 25 clinical subjects, the most common diagnosis was UIP/IPF (7/25). Other diagnoses are displayed in Figure 2. Representative examples of cryobiopsies from several diagnoses are shown in Figure 3. Among subjects found to have UIP, hallmark features including temporal heterogeneity, microscopic honeycomb cysts and fibroblastic foci from cryobiopsy specimens are demonstrated in Figure 4.

Safety outcomes are summarized in Table 3. During the study period, there were no pneumothoraces or bleeding requiring additional intervention. One day after discharge, one outpatient was admitted to the hospital for overnight observation for hemoptysis that spontaneously resolved. One inpatient was observed one night in the intensive care unit for post-procedural hypoxemia that returned to baseline oxygen requirement within 12 hours. No patients required intubation or use of noninvasive ventilation after the procedure.

**Table 3:** Safety outcomes.

	Outpatients	Inpatients
	(n = 33)	(n = 4)
Pneumothorax	0	0
Bleeding	0	0
Hospitalization after procedure	1	n/a
ICU admission	0	1
Intubation/Mechanical Ventilation	0	0
ICU LOS (days)	n/a	1
Hospital LOS (days)	1	3.75 (3–4)

Data are presented as number of subjects or mean (range) where appropriate.

## Discussion

In this series of twenty-five patients with DPLD who required lung biopsy, in 20 cases (80%) lung tissue obtained from a bronchoscopic cryobiopsy, when combined with clinical and radiographic information, was sufficient to establish a diagnosis. This represents a striking improvement in diagnostic yield compared to historical studies evaluating the use of traditional forceps transbronchial biopsies which report diagnostic yield in approximately 30% of cases [7], [8], [9].

When compared to surgical lung biopsy, performed either via mini-thoracotomy or using video-assisted thoracic surgery (VATS), bronchoscopic cryobiopsy offers several advantages. As we report here, this procedure can be safely performed in the outpatient setting with low risk of need for hospitalization. The risk of general anesthesia, placement of a double-lumen endotracheal tube and single lung ventilation are avoided with bronchoscopic cryobiopsy. Patient discomfort from incisional and/or chest tube site pain is eliminated. We did not observe any pneumothoraces following bronchocopic cryobiopsy so although we cannot comment on the risk of prolonged air leak or bronchopleural fistula, this suggests it is likely reduced compared to surgical lung biopsy. A previous series reported pneumothorax rates similar to that of transbronchial forceps biopsy [10].

There are several reasons why the diagnostic yield of cryobiopsies may exceed that or forceps biopsies. First, cryobiopsy samples appear to be substantially larger in size than forceps biopsies; in this series the mean longest dimension of cryobiopsy samples was 8.7 mm, 3–5 times larger than those typically obtained using forceps [10], [12]. This is accompanied by a substantial increase in biopsy area; the mean biopsy area we report is similar to that observed in a study of lung transplant patients who underwent bronchoscopic cryobiopsy [12]. This increase in area offers more contiguous alveolated lung for characterization of spatial/temporal heterogeneity which is critical for establishing a diagnosis of UIP/IPF. Similarly, samples more likely contain multiple small airways for evaluation of suspected bronchiolitis. As has previously been reported [10], [12], crush artifact caused by the biopsy procedure is minimized as the freezing process does not appear to alter lung architecture.

The primary safety concern of bronchoscopic cryobiopsy is bleeding risk, as the bronchoscope must be withdrawn from the airway to remove the biopsy specimen from the cryoprobe, thus an immediate bronchoscope wedge to tamponade bleeding cannot be achieved. To minimize this risk, all antiplatelet and anticoagulant medications were discontinued at least 7 days before the cryobiopsy procedure. Whenever possible, biopsies were taken from dependent areas of the lung. Once the biopsy was removed from the probe, the bronchoscope was quickly returned to the airway and wedged in the biopsied segment for three minutes to tamponade bleeding and then was slowly withdrawn. We did not observe any bleeding requiring additional intervention or blood transfusion; typical blood loss was 15 milliliters or less.

This study, by nature of its retrospective design, has several limitations. The sample size we report is small and from a single-center where all procedures were performed by an experienced interventional pulmonologist. Therefore, the diagnostic yield and safety outcomes might differ among operators with variable experience. It is possible that referral bias may have enriched the study population for patients/conditions more likely to have “diagnostic” cryobiopsies. Most patients who underwent bronchoscopic cryobiopsy had abnormal pulmonary function tests demonstrating mild-to-moderate restriction with a reduced diffusing capacity for carbon monoxide (DLCO) and “atypical” imaging that did not meet ATS/ERS criteria for definite or probable UIP. While this possibility of a referral bias cannot be completely excluded, all subjects met criteria for referral for surgical lung biopsy at our institution. Two of 25 subjects were found to have malignancy, and 0/25 had sarcoidosis, the two conditions for which traditional transbronchial forceps biopsy is of highest yield, thus we believe the subjects are a representative sample of patients with DPLD referred to a specialty interstitial lung disease clinic at a tertiary care center. In all patients, biopsies were taken from areas of the lung that appeared radiographically abnormal, similar to the approach employed for transbronchial forceps biopsies at this institution wherein two separate affected areas are customarily sampled. The majority of biopsy specimens were taken from lower lobes, and it is possible that this strategy may be subject to sampling error as upper lobes may have been biopsied less frequently than would have been the case with surgical lung biopsy. Blinding of the clinical biopsy samples for pathological review was not possible given the retrospective nature of the study. Finally, no cryobiopsies were performed on subjects with substantial baseline oxygen requirements (>4 Lpm) or patients with respiratory failure requiring mechanical ventilation, thus the safety and yield of bronchoscopic cryobiopsies in this setting is not known.

We believe that this technique has the potential to dramatically change practice in the evaluation of patients with DPLD. For many patients with DPLD and “atypical” imaging findings, bronchoscopic cryobiopsy offers a reasonable likelihood of establishing a diagnosis while avoiding the morbidity of surgical lung biopsy. Further study will be necessary to determine the comparative cost-effectiveness of bronchoscopic cryobiopsy compared to surgical lung biopsy, however we anticipate cryobiopsy may offer substantial cost savings.

## Conclusions

The evaluation of diffuse parenchymal lung disease is complex, and in many patients, lung biopsy is necessary to establish a diagnosis. Bronchoscopic cryobiopsy can be performed safely (in experienced hands) in patients with DPLD and offers promise of achieving adequate tissue for diagnosis in many patients without the risk and morbidity of surgical lung biopsy. As experience with this technique increases, bronchoscopic cryobiopsy holds promise to become the preferred, first-line approach to lung biopsy in the diagnostic approach to the patient with DPLD.

## References

[1] Raghu G, Collard HR, Egan JJ, Martinez FJ, Behr J, et al (2011) An official ATS/ERS/JRS/ALAT statement: idiopathic pulmonary fibrosis: evidence-based guidelines for diagnosis and management. Am J Respir Crit Care Med 183: 788–824. 21471066 10.1164/rccm.2009-040GL PMC5450933

[2] Noble PW, Albera C, Bradford WZ, Costabel U, Glassberg MK, et al (2011) Pirfenidone in patients with idiopathic pulmonary fibrosis (CAPACITY): two randomised trials. Lancet 377: 1760–1769. 21571362 10.1016/S0140-6736(11)60405-4

[3] Richeldi L, Costabel U, Selman M, Kim DS, Hansell DM, et al (2011) Efficacy of a tyrosine kinase inhibitor in idiopathic pulmonary fibrosis. N Engl J Med 365: 1079–1087. 21992121 10.1056/NEJMoa1103690

[4] Krasna MJ, White CS, Aisner SC, Templeton PA, McLaughlin JS (1995) The role of thoracoscopy in the diagnosis of interstitial lung disease. Ann Thorac Surg 59: 348–351. 7847948 10.1016/0003-4975(94)00844-w

[5] Utz JP, Ryu JH, Douglas WW, Hartman TE, Tazelaar HD, et al (2001) High short-term mortality following lung biopsy for usual interstitial pneumonia. Eur Respir J 17: 175–179. 11334116 10.1183/09031936.01.17201750

[6] Poletti V, Patelli M, Poggi S, Bertanti T, Spiga L, et al (1988) Transbronchial lung biopsy and bronchoalveolar lavage in diagnosis of diffuse infiltrative lung diseases. Respiration 54 Suppl 166–72. 3231906 10.1159/000195479

[7] Shim HS, Park MS, Park IK (2010) Histopathologic findings of transbronchial biopsy in usual interstitial pneumonia. Pathol Int 60: 373–377. 20518887 10.1111/j.1440-1827.2010.02528.x

[8] Berbescu EA, Katzenstein AL, Snow JL, Zisman DA (2006) Transbronchial biopsy in usual interstitial pneumonia. Chest 129: 1126–1131. 16685001 10.1378/chest.129.5.1126 PMC2094131

[9] Tomassetti S, Cavazza A, Colby TV, Ryu JH, Nanni O, et al (2012) Transbronchial biopsy is useful in predicting UIP pattern. Respir Res 13: 96. 23107232 10.1186/1465-9921-13-96 PMC3499172

[10] Babiak A, Hetzel J, Krishna G, Fritz P, Moeller P, et al (2009) Transbronchial cryobiopsy: a new tool for lung biopsies. Respiration 78: 203–208. 19246874 10.1159/000203987

[11] Pajares V, Torrego A, Puzo C, Lerma E, Gil De Bernabe MA, et al (2010) [Transbronchial lung biopsy using cryoprobes]. Arch Bronconeumol 46: 111–115. 19939546 10.1016/j.arbres.2009.09.012

[12] Yarmus L, Akulian J, Gilbert C, Illei P, Shah P, et al (2013) Cryoprobe transbronchial lung biopsy in patients after lung transplantation: a pilot safety study. Chest 143: 621–626. 23328889 10.1378/chest.12-2290

[13] Pritchett JM, Kropski JA, Mason WR, Lancaster LH, Lawson WE, et al. (2013) Bronchoscopic cryobiopsy for the diagnosis of diffuse parenchymal lung disease. Am J Respir Crit Care Med American Thoracic Society International Conference Abstracts: A5978–A5978. 10.1371/journal.pone.0078674 PMC3827078 24265706

[14] Kropski JA, Lawson WE, Young LR, Blackwell TS (2013) Genetic studies provide clues on the pathogenesis of idiopathic pulmonary fibrosis. Dis Model Mech 6: 9–17. 23268535 10.1242/dmm.010736 PMC3529334

[15] Rosas IO, Ren P, Avila NA, Chow CK, Franks TJ, et al (2007) Early interstitial lung disease in familial pulmonary fibrosis. Am J Respir Crit Care Med 176: 698–705. 17641157 10.1164/rccm.200702-254OC PMC1994234

